# *vanI*: a novel d-Ala-d-Lac vancomycin resistance gene cluster found in *Desulfitobacterium hafniense*

**DOI:** 10.1111/1751-7915.12139

**Published:** 2014-07-05

**Authors:** Thomas Kruse, Mark Levisson, Willem M de Vos, Hauke Smidt

**Affiliations:** 1 Laboratory of Microbiology, Wageningen UniversityWageningen, HB 6703, The Netherlands; 2 Department of Basic Veterinary Medicine, Department of Bacteriology and Immunology, University of HelsinkiHelsinki, FI 00014, Finland

## Abstract

The glycopeptide vancomycin was until recently considered a drug of last resort against Gram-positive bacteria. Increasing numbers of bacteria, however, are found to carry genes that confer resistance to this antibiotic. So far, 10 different vancomycin resistance clusters have been described. A chromosomal vancomycin resistance gene cluster was previously described for the anaerobic *Desulfitobacterium hafniense* Y51. We demonstrate that this gene cluster, characterized by its d-Ala-d-Lac ligase-encoding *vanI* gene, is present in all strains of *D*. *hafniense*, *D. chlororespirans* and some strains of *Desulfosporosinus* spp. This gene cluster was not found in vancomycin-sensitive *Desulfitobacterium* or *Desulfosporosinus* spp., and we show that this antibiotic resistance can be exploited as an intrinsic selection marker for *Desulfitobacterium hafniense* and *D. chlororespirans*. The gene cluster containing *vanI* is phylogenetically only distantly related with those described from soil and gut bacteria, but clusters instead with vancomycin resistance genes found within the phylum *Actinobacteria* that include several vancomycin-producing bacteria. It lacks a *vanH* homologue, encoding a D-lactate dehydrogenase, previously thought to always be present within vancomycin resistance gene clusters. The location of *vanH* outside the resistance gene cluster likely hinders horizontal gene transfer. Hence, the vancomycin resistance cluster in *D*. *hafniense* should be regarded a novel one that we here designated *vanI* after its unique d-Ala-d-Lac ligase.

## Introduction

Antibiotic resistance is a two-edged sword. On one hand, it has proven extremely useful, as a convenient selection marker for biotechnological applications and a fast way to eliminate the susceptible fraction from enrichment cultures (Löffler *et al*., 2005; Schweizer, 2008). On the other hand, the spread of antibiotic resistance among bacteria can lead to multi-resistant pathogens, making their inhibition one of the most serious challenges in modern healthcare (Davies and Davies, 2010).

The glycopeptides vancomycin and teicoplanin are widely used antibiotics for treatment of infections with Gram-positive bacteria. They act by binding to the d-alanyl-d-alanine (d-Ala-d-Ala) terminus of intermediates in peptidoglycan formation, thereby inhibiting cell wall cross-linking (Courvalin, 2006). Since the late 1980s, vancomycin-resistant pathogens have emerged in hospitals worldwide. Vancomycin resistance results from the production of modified peptidoglycan precursors terminating in either d-alanyl-d-lactate (d-Ala-d-Lac) or d-alanyl-d-serine (d-Ala-d-Ser) to which vancomycin exhibits low binding affinities. Vancomycin resistance is classified into clusters based on the DNA sequence of the ligase gene *vanA* and its homologues that encode the key enzyme in the synthesis of d-Ala-d-Lac or d-Ala-d-Ser. Currently, 10 different types of vancomycin resistance gene clusters have been identified. d-Ala-d-Lac type resistance is associated with clusters *vanA*, *vanB*, *vanD*, *vanF and vanM*, whereas d-Ala-d-Ser resistance is linked to clusters *vanC*, *vanE*, *vanG*, *vanL and vanN* (Fraimow *et al*., 2005; Depardieu *et al*., 2007; Boyd *et al*., 2008; Xu *et al*., 2010; Lebreton *et al*., 2011). The *vanA* cluster also confers a high level of resistance to teicoplanin, whereas the other gene clusters of the d-Ala-d-Lac type give less or no resistance to teicoplanin (Kalan *et al*., 2009; Xu *et al*., 2010).

**Figure 1:**
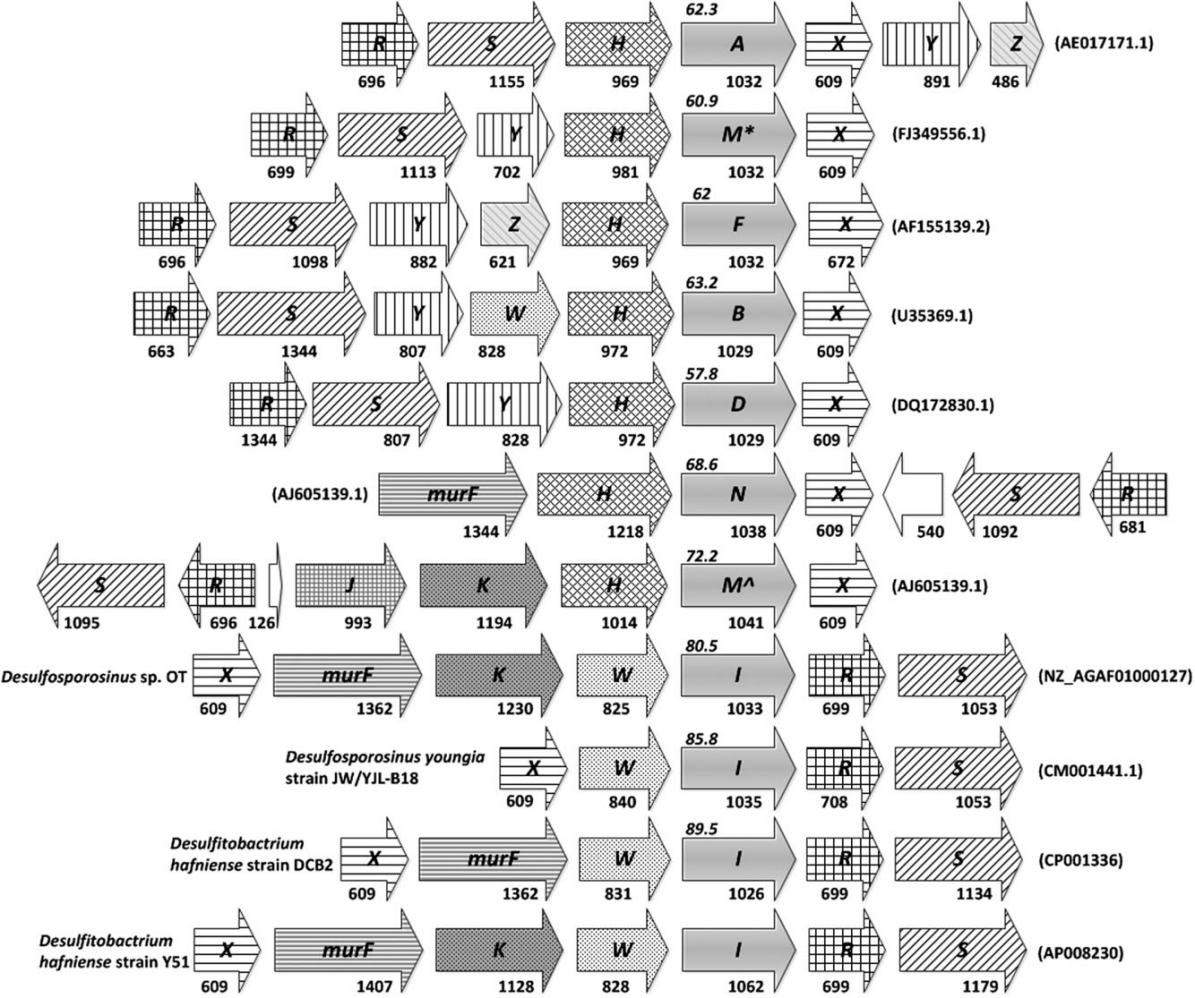
Alignment of vancomycin resistance gene clusters representing the currently known *van* gene clusters (see also Fig. 3). Arrows show the direction of transcription, while arrow size indicates gene size. Letters inside arrows denote gene names with the ‘*van*’ prefix removed. Empty arrows are hypothetical genes. Light grey arrows represent the d-Ala-d-Lac ligases giving name to the gene clusters. Numbers under arrows indicate gene size in bp, and numbers in *italics* above d-Ala-d-Lac ligases show percentage amino acid identity to *VanI* from *Desulfitobacterium hafniense* strain Y51. *VanM according to (Xu *et al*., 2010), ^∧^*VanM* according to (Aminov and Mackie, 2007). Note that *vanI* previously was referred to as *vanA* by Kalan and coauthors (Kalan *et al*., 2009). Accession numbers are given in parentheses.

Three genes are considered essential for d-Ala-d-Lac-mediated vancomycin resistance: *vanH*, *vanA* and homologues, and *vanX*. These are found in three genes forming the *vanHAX* core that is conserved across pathogenic bacteria, glycopeptide antibiotic producers and other environmental bacteria (Fig. 1) (Marshall *et al*., 1998, Kalan *et al*., 2009 and the references cited therein). The *vanH* gene encodes a d-lactate dehydrogenase that converts pyruvate to d-lactate, *vanA* and homologues encode an ATP-dependent depsipeptide ligase involved in the synthesis of d-Ala-d-Lac, and *vanX* encodes a dipeptidase that cleaves d-Ala-d-Ala present in the cell, thereby enriching the cell wall in d-Ala-d-Lac. These key genes are found together with a flexible pool of accessory genes, such as those encoding D,D-carboxypeptidases and regulatory proteins (Fig. 1) (Depardieu *et al*., 2007; Hong *et al*., 2008).

*Desulfitobacterium* spp. include strictly anaerobic bacteria that have mainly been isolated from environments contaminated with halogenated organic compounds. Most strains of *Desulfitobacterium* spp. isolated to date are able to degrade one or more halogenated compounds by organohalide respiration (Villemur *et al*., 2006). One of the few exceptions is *D. hafniense* DP7, which was isolated from a human faecal sample (van de Pas *et al*., 2001). Previously, a vancomycin resistance gene cluster was identified in the tetrachloroethene (PCE) dehalogenating bacterium *Desulfitobacterium hafniense* Y51 (Kalan *et al*., 2009). The predicted *vanA* gene was not found in the usual context of three genes forming the *vanHAX* core, but rather was present in a cluster consisting of seven genes, referred to as *vanXmurFvanKWARS* (Fig. 1) (Kalan *et al*., 2009). This gene cluster does not contain a *vanH* gene, unlike clusters previously described for vancomycin-resistant bacteria. Functional expression of the genes in *D. hafniense* Y51 was confirmed by resistance to vancomycin, with a minimal inhibitory concentration (MIC) for growth of 64 μg/ml (Kalan *et al*., 2009).

In the present study, we investigated the distribution and phylogenetic affiliation of vancomycin resistance among *Desulfitobacterium* spp. of environmental and human origin. In addition, it was investigated whether vancomycin could be successfully used as a selective agent for isolation of *Desulfitobacterium* spp. from enrichment cultures.

## Results

### d-Ala-d-Lac ligases-in Desulfitobacterium spp

**Figure 2:**
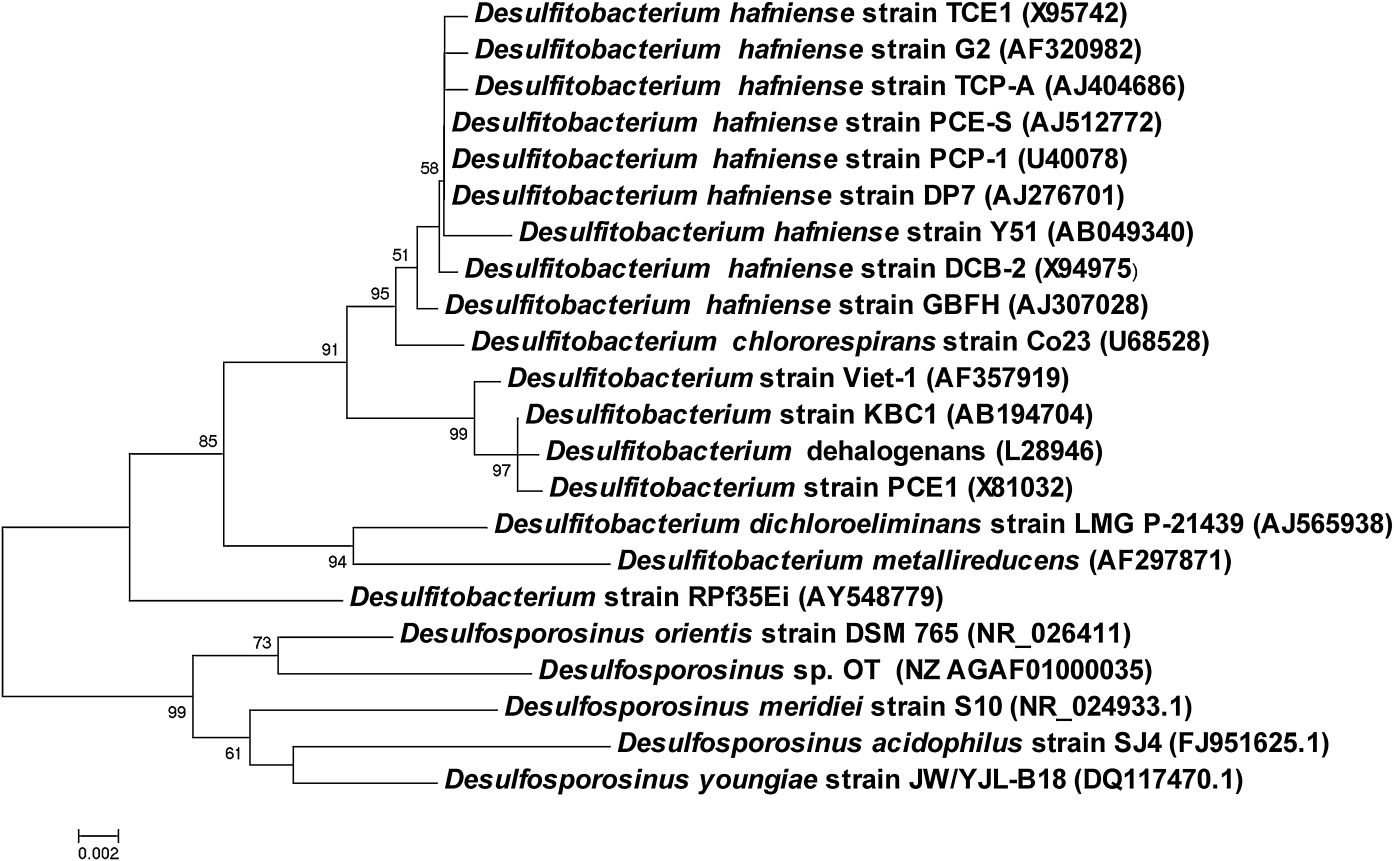
16S rRNA gene-based phylogenetic tree of *Desulfitobacterium* spp. and *Desulfosporosinus* spp. Sequences were aligned with muscle (Edgar, 2004), and a neighbour-joining tree was constructed and validated with 1000 bootstraps analysis using the mega5 software package (Tamura *et al*., 2011). Bootstrap values higher than 50% are given at corresponding nodes in the tree. The reference bar indicates 0.2% base substitutions per site. GenBank accession numbers of sequences used in this analysis are given in parentheses.

Chromosomal DNA was isolated from 13 *Desulfitobacterium* spp. strains, covering the majority of the currently described species of this genus. The DNA sequences of the two d-Ala-d-Lac ligase-encoding genes from *D. hafniense* Y51 (DSY3690) and *D. hafniense* DCB-2 (Dhaf_1664) were used to design primers for the detection of homologous d-Ala-d-Lac ligase genes in other *Desulfitobacterium* spp. strains. Amplicons of the expected size of 987 bp were obtained for nine of the 13 tested strains (Table 1). All nine polymerase chain reaction (PCR)-positive isolates were strains of *D. hafniense*, with the exception of *D. chlororespirans*, which is the closest relative of *D. hafniense* based on 16S rRNA gene similarity (Fig. 2). Sequence analysis of the obtained PCR products confirmed that they encode d-Ala-d-Lac ligases (data not shown).

**Table 1:** *Desulfitobacterium* spp. and *Desulfosporosinus* spp. strains used in this study

#	Strain	Presence of *vanI*	Vancomycin MIC [μg/ml]	Teicoplanin MIC [μg/ml]
(a) *Desulfitobacterium* spp.				
1	*D. hafniense* Y51^a^	Yes (AP008230.1)	> 250	> 50 < 75
2	*D. hafniense* TCE1^b^	Yes (HQ433581)	> 250	> 50 < 75
3	*D. hafniense* DP7 (DSM^c^ 13498)	Yes (HQ433580)	> 150 < 250	> 25 < 50
4	*D. hafniense* DCB-2 (DSM^c^ 10664)	Yes (CP001336.1)	> 150 < 250	> 25 < 50
5	*D. hafniense* PCE-S (DSM^c^ 14645)	Yes (HQ433585)	> 250	> 25 < 50
6	*D. hafniense* PCP-1 (DSM^c^ 12420)	Yes (HQ433583)	> 150 < 250	> 50 < 75
7	*D. hafniense* TCP-A (DSM^c^13557)	Yes (HQ433582)	> 250	> 25 < 50
8	*D. hafniense* G2 (DSM^c^ 16228)	Yes (HQ433584)	> 250	> 50 < 75
9	*D. chlororespirans* (DSM^c^ 11544)	Yes (HQ433586)	> 250	> 50 < 75
10	*D. dehalogenans* (DSM^c^ 9161)	No	< 1	< 1
11	*D. sp*. PCE1 (DSM^c^ 10344)	No	< 1	< 1
12	*D. metallireducens* (DSM^c^ 15288)	No	ND	ND
13	*D. dichloroeliminans* DCA1^d^	No	ND	ND
(b) *Desulfosporosinus* spp.				
14	*D. youngia* (DSM^c^ 17734)	Yes (2508507521)^e^	> 10 < 25	> 5 < 10
15	*D. orientis* (DSM^c^ 765)	No	< 1	< 1
16	*D. meridiei* (DSM^c^ 13257)	No	< 1	< 1
17	*D. acidiphilus* (DSM^c^ 22704)	No	< 1	< 1

a Received as a kind gift from Masatoshi Goto, Department of Bioscience and Biotechnology, Faculty of Agriculture, Kyushu University, Japan.

b Received as a kind gift from Jan Dirk van Elsas, Department of Microbial Ecology, University of Groningen, The Netherlands.

c DSM, Leibniz Institute DSMZ – German Collection of Microorganisms and Cell Cultures, Braunschweig, Germany.

d Received as a kind gift from Nico Boon, Laboratory of Microbial Ecology and Technology, Ghent University, Belgium.

e JGI gene ID.

Accession numbers are given in parentheses.

**Figure 3:**
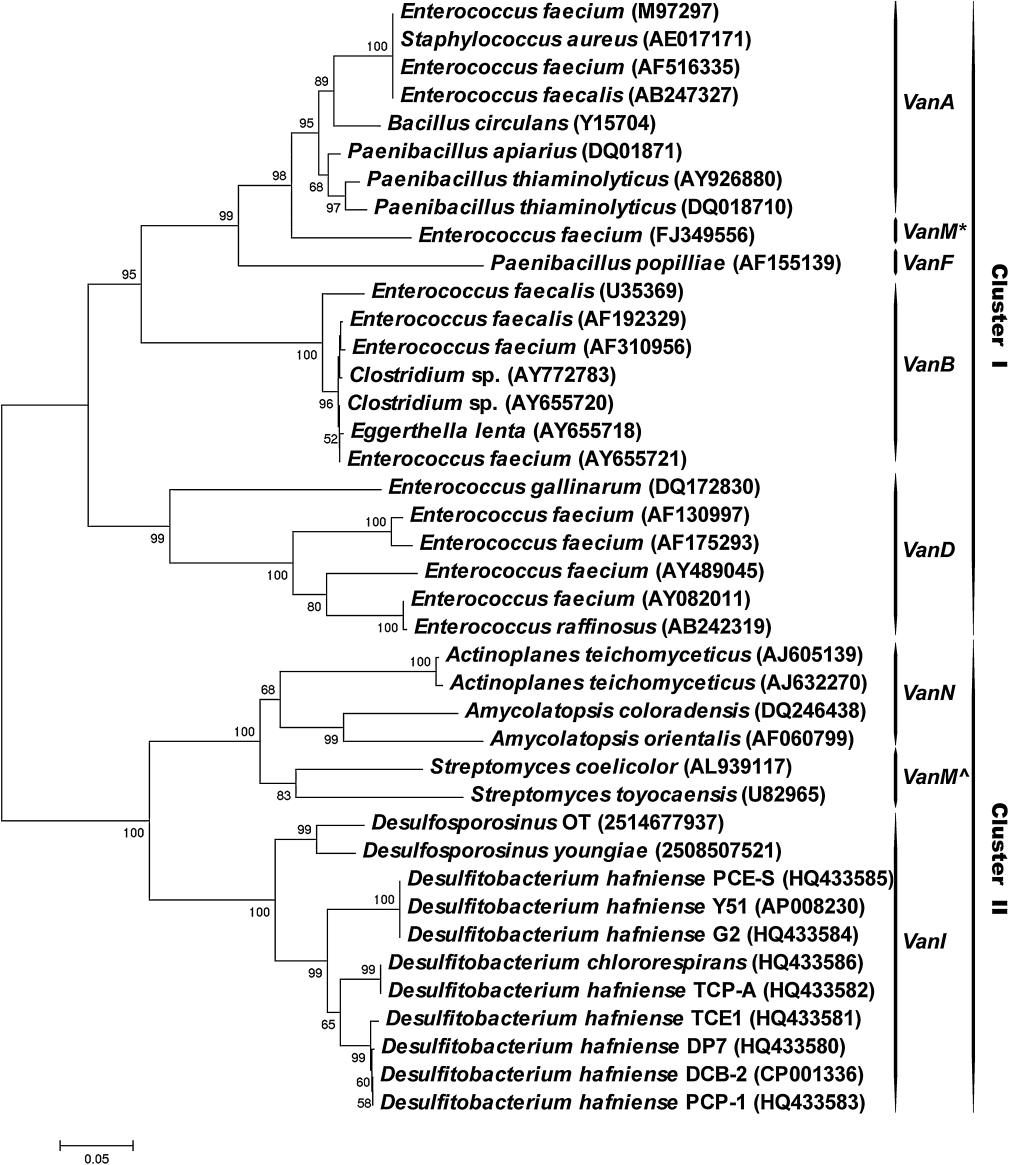
Phylogenetic tree based on a 512 bp fragment of d-Ala-d-Lac ligase encoding genes representing the currently known *Van* gene clusters of the d-Ala-d-Lac type. Corresponding reference sequences (Aminov and Mackie, 2007; Xu *et al*., 2010), *vanI* sequences obtained in this study and sequences retrieved from genome data sets (Teo *et al*., 2011; Pester *et al*., 2012; Markowitz *et al*., 2012a) were aligned with muscle (Edgar, 2004), and a neighbour-joining tree was constructed and validated with 1000 bootstraps analysis using the mega5 software package (Tamura *et al*., 2011). *VanM according to (Xu *et al*., 2010), ^∧^*VanM* according to (Aminov and Mackie, 2007). Bootstrap values higher than 50% are given at corresponding nodes in the tree. The reference bar indicates 5% base substitutions per site. GenBank accession numbers or JGI gene IDs of sequences used in this analysis are given in parentheses. Definition of *van* gene clusters was adapted from (Aminov and Mackie, 2007).

### Phylogenetic analysis

Previously, a phylogenetic analysis was performed using a data set of concatenated open reading frames (ORFs) comprising *vanH*, *vanA* and its homologues, and *vanX* (Aminov and Mackie, 2007). This analysis showed a very early branching event, leading to the formation of two large clusters, one of which consists of genes associated with vancomycin-resistant enterobacteria and other vancomycin-resistant gut and soil bacteria (cluster I). The second cluster consists of members of the *Actinobacteria*, some of which have been shown to produce vancomycin (cluster II). Furthermore, this phylogenetic analysis suggested that *vanHAX* and homologous gene clusters behave as a single evolutionary unit (Aminov and Mackie, 2007).

We constructed a phylogenetic tree using the nucleotide sequences of 512 bp fragments corresponding to part of the d-Ala-d-Lac ligase genes obtained in this study. This tree showed essentially the same clustering as when using the sequences of the entire *vanHAX* and homologous gene clusters (data not shown), indicating that the sequence fragments obtained in this study are sufficient for robust phylogenetic analysis. We then included in the phylogenetic tree the 512 bp sequence fragments obtained from *Desulfitobacterium* spp., closely related *Desulfosporosinus* spp., and sequences from the newly described *vanM* cluster (Abicht *et al*., 2011; Teo *et al*., 2011), resulting in a tree containing representative sequences from all currently described vancomycin resistance clusters of the d-Ala-d-Lac type (Fig. 3). The phylogenetic tree shows two main and deep-branching clusters. Cluster I consists of *vanA*, vanM*, *vanF*, *vanB* and *vanD*, while cluster II consists of *vanM*^∧^, *vanN*, and a separate subcluster comprising all d-Ala-d-Lac ligase-encoding sequences obtained from *Desulfitobacterium* spp. and *Desulfosporosinus* spp. (Fig. 3). Although the sequences obtained from glycopeptide producing *Actinobacteria*, *Desulfitobacterium* spp. and *Desulfosporosinus* spp. belong to cluster II, it can be assumed that desulfitobacteria do not produce vancomycin, as no vancomycin synthesis pathway was identified in the two published genomes of *Desulfitobacterium hafniense* DCB-2 and Y51 (Nonaka *et al*., 2006; Kim *et al*., 2012), nor in the available draft genome sequence of strain DP7 (AFZX00000000.1).

Sequences obtained from *Desulfitobacterium* spp. and *Desulfosporosinus* spp. were found to be more similar to those of the d-Ala-d-Lac ligases belonging to cluster II than to any of the sequences from cluster I (Supporting Information Table S1). Based on these results, it is clear that the d-Ala-d-Lac ligase gene found in *Desulfitobacterium* spp. and *Desulfosporosinus* spp. does not belong to the *vanA* cluster, but instead is part of a distinct vancomycin resistance cluster, which we propose to name *vanI* after its unique d-Ala-d-Lac ligase.

### Presence of *vanI* outside the *Desulfitobacterium* genus

BlastP analysis against all sequenced bacterial genomes identified *vanI* homologues in the genomes of both *Desulfosporosinus youngiae* JW/Y JL-B18 and *Desulfosporosinus* sp. OT (Fig. 3), but not in *Desulfosporosinus orientis* Singapore I, *D. meridiei* S10 or *D. acidiphilus* SJ4 (Abicht *et al*., 2011; Pester *et al*., 2012; Markowitz *et al*., 2012a). We did not detect *vanI*-like genes in any of the metagenomes publicly available in the JGI IMG/M repository (as of 4 February 2013), demonstrating that *vanI* genes are not widespread in the environment or the human gut.

### Architecture of the *vanI* gene cluster

Inspection of the genomic sequences of *Desulfitobacterium* spp*. and Desulfosporosinus* spp. revealed the *vanX_I_murF_I_vanK_I_W_I_IR_I_S_I_* (*vanXmurFvanKWARS* in Kalan *et al*., 2009) gene cluster to be conserved (Fig. 1). However, it should be noted that *vanK_I_*, encoding the enzyme adding the branched amino acid(s) to the stem pentapeptide of peptidoglycan precursors (Hong *et al*., 2005), was only found in *Desulfitobacterium hafniense* Y51 and *Desulfosporosinus* sp. OT. An interesting feature of the *vanI* gene cluster is the location of the gene encoding the d-Lac dehydrogenase (*vanH*) outside the vancomycin resistance gene cluster. Usually, *vanH* genes are part of the three genes *vanHAX* or homologues that form the core of vancomycin resistance gene clusters (Hong *et al*., 2008). Previously, four genes have been proposed as potential *vanH*-encoding genes (DSY0996, DSY1673, DSY3442 and DSY4020) in the genome of *D. hafniense* Y51 (Kalan *et al*., 2009). The homologues of these four genes were also found in the genomes of *D. hafniense* DCB-2 (Dhaf_1344, Dhaf_1980, Dhaf_2079 and Dhaf_2820) and *D. hafniense* DP7 (data not shown). The complete genomes of *D. hafniense* Y51 and DCB-2 (Nonaka *et al*., 2006; Kim *et al*., 2012) were aligned and inspected for signs of horizontal transfer of the vancomycin resistance gene cluster. No genes encoding integrases or transposases, nor large deviations in dinucleotide frequency or GC content, were found in the vicinity of the vancomycin resistance gene clusters, suggesting that they are not located on mobile genomic islands.

### Vancomycin and teicoplanin susceptibility

In total, 15 strains belonging to three different species of *Desulfitobacterium* and four species of *Desulfosporosinus* were tested for growth on plates containing different concentrations of vancomycin or teicoplanin. All strains for which a *vanI* gene was detected are resistant to vancomycin and teicoplanin (Table 1). Strains of *Desulfitobacterium* spp. showed high level of resistance with MIC values > 150 μg/ml vancomycin and > 25 ug/ml teicoplanin (Table 1). The two strains that tested negative for *vanI* genes, namely *D. dehalogenans* and the closely related strain PCE1, are highly susceptible to both vancomycin and teicoplanin, with MIC values < 1 μg/ml. The same was the case for the tested members of the *Desulfosporosinus* genus. *Desulfosporosinus youngia*, whose genome contains a *vanI* gene cluster, is resistant to vancomycin and teicoplanin, although their MICs were approximately tenfold lower than those observed for *Desulfitobacterium* spp. The three tested *Desulfosporosinus* spp. strains lacking a *vanI* gene are all susceptible to both vancomycin and teicoplanin (Table 1).

### Vancomycin as selective agent

Previous experiments with cultures of the PCE-dehalogenating strain *D. hafniense* TCE1, received from collaborators as well as from Deutsche Sammlung von Mikroorganismen und Zellkulturen (DSMZ), led us to suspect that the cultures contained a microbial contaminant. Partial 16S rRNA gene sequencing confirmed that the *D. hafniense* TCE1 culture was contaminated with a *Sedimentibacter* sp. (97% 16S rRNA gene sequence identity to *Sedimentibacter* sp. C7, AY766466.1) (data not shown). *Sedimentibacter* spp. are gram-positive, members of the *Firmicutes*, and as a consequence likely susceptible to vancomycin (Breitenstein *et al*., 2002). We, therefore, decided to test vancomycin as a potential selective agent for the elimination of the *Sedimentibacter* contaminant.

A contaminated *D. hafniense* TCE1 culture was first grown in liquid medium with 20 mM lactate and 10 mM PCE as electron donor and acceptor, respectively, in order to preselect for the dechlorinating *Desulfitobacterium*. Subsequently, an early exponential growth phase culture was plated on medium containing 40 mM pyruvate and supplemented with 50 μg/ml vancomycin. After 1 week, only one colony type was observed. Two colonies were picked and grown in liquid medium containing 20 mM lactate and 10 mM PCE, supplemented with 50 μg/ml vancomycin and subsequently transferred to liquid medium containing 40 mM pyruvate and no vancomycin. This resulted in a pure culture of *D. hafniense* TCE1 without the presence of any *Sedimentibacter*, as confirmed by screening of a general bacterial 16S rRNA gene clone library with primers specific for *Desulfitobacterium* spp.

## Discussion

In this contribution, the distribution of vancomycin resistance among *Desulfitobacterium* spp. and *Desulfosporosinus* spp. is described. Nine of the 13 tested strains of *Desulfitobacterium* spp. display high levels of vancomycin resistance, whereas one out of four strains of *Desulfosporosinus* spp. was found moderately vancomycin-resistant (Table 1). Previously, a vancomycin MIC value of 64 μg/ml was reported for *D. hafniense* Y51 (Kalan *et al*., 2009), which is considerably lower than the values obtained in this study (Table 1). A possible explanation for the higher MIC values could be the differences in the plating medium, and a protocol between this study and the study of Kalan and colleagues (Kalan *et al*., 2009). It was, for instance, demonstrated that the growth on plates of *Desulfitobacterium dehalogenans* was much improved when 0.8% of highly purified agar was used instead of 1.5% agar of standard purity (Smidt *et al*., 1999).

Phylogenetic analysis of predicted d-Ala-d-Lac ligase encoding genes showed that those obtained from *Desulfitobacterium* spp. together with *Desulfosporosinus* spp. belong to cluster II where they form a distinct branch, next to genes retrieved from glycopeptide-producing members of the phylum *Actinobacteria* (Fig. 3). It has often been speculated that antibiotic resistance, including vancomycin resistance, was developed as a mechanism to prevent suicide by antibiotic-producing bacteria. According to this hypothesis, resistance genes then spread to soil bacteria before, finally, via an intermediate host bacterium, ending up in enterobacteria and other gut bacteria (Benveniste and Davies, 1973; Nonaka *et al*., 2006; D’Costa *et al*., 2007; Forsberg *et al*., 2012). The phylogenetic analysis of Aminov and Mackie (2007) suggested an early diversification between cluster I (soil and gut bacteria) and cluster II (glycopeptide-producing *Actinobacteria*), with no later exchange of genes between these (Aminov and Mackie, 2007). The vancomycin resistance genes found in *Desulfitobacterium* spp. and *Desulfosporosinus* spp. form a distinct group in cluster II, indicating that they have been acquired from vancomycin producers at a later stage than those belonging to cluster I (Fig. 3). Therefore, we propose that the vancomycin resistance gene cluster found in *Desulfitobacterium* spp. and *Desulfosporosinus* spp. should be considered as a novel cluster, designated *vanI* rather than a variant of the *vanA* type as initially suggested for *D. hafniense* Y51 (Kalan *et al*., 2009).

The basic structure of the *vanX_I_murF_I_vanK_I_W_I_IR_I_S_I_* gene cluster was found to be conserved in the published genomes of *D. hafniense* strain Y51, DCB-2, *Desulfosporosinus* sp. OT and *Desulfosporosinus youngiae* (Fig. 1) (Nonaka *et al*., 2006; Abicht *et al*., 2011; Kim *et al*., 2012; Pester *et al*., 2012). The only exceptions to this conserved structure found so far are that *murF_I_* was not found in *Desulfosporosinus youngiae*, and *vanK_I_* is only present in *D. hafniense* Y51 and *Desulfosporosinus* sp. OT (Fig. 1). A characteristic feature of the *VanI* vancomycin resistance gene cluster is the absence of a gene encoding the d-Lac dehydrogenase (VanH). This is the first vancomycin resistance gene cluster, where the *vanH* gene is located on the chromosome outside the resistance cluster.

Desulfitobacteria have been found both in the environment and in the human gut (Villemur *et al*., 2006). Hence, it is tempting to speculate that they potentially could act as a shuttle for vancomycin resistance between the environment and the gut microbiota. However, no indications were found in the available *Desulfitobacterium* spp. genomes that the vancomycin resistance genes are or have been mobile. The location of *vanH* homologues elsewhere in the genome, rather than in the vancomycin resistance cluster, likely acts as a barrier against horizontal transfer of this phenotype.

The genera *Desulfitobacterium* and *Desulforosporosinus* are phylogenetically closely related, both belonging to the family *Peptococcaceae* (Fig. 2) (Spring and Rosenzweig, 2006). It seems likely that the *vanI* gene cluster was acquired by a common ancestor of *Desulfitobacterium* spp. and *Desulforosporosinus* spp. based on the very high sequence similarity between their *vanI* sequences, and the characteristic location of *vanH* homologues outside the resistance gene cluster (Figs 1 and 3). It is interesting to note that *Streptomyces coelicolor vanM*^∧^ is the d-Ala-d-Lac ligase encoding homologue showing highest identity to *vanI* (Supporting Information Table S1). This is also the only other vancomycin resistance gene cluster in which *vanK* has been found (Hong *et al*., 2004; 2008). A *Streptomyces coelicolor* Δ*vanK* mutant was shown to be unable to grow in the presence of vancomycin (Hong *et al*., 2005). In contrast, *vanK* is not essential for vancomycin resistance in *Desulfitobacterium* spp., as both *Desulfitobacterium hafniense* DP7 and DCB-2 are resistant to vancomycin, despite not encoding a *vanK_I_*. Inspecting the genomes of *Desulfitobacterium hafniense* DCB-2 and DP7 confirmed that *vanK* unlike *vanH* is not located elsewhere in the genomes. *Streptomyces* spp. do not share a recent common ancestor with *Desulfitobacterium* spp. or *Desulfosporosinus* spp.; thus, it is plausible that the *vanI* gene cluster was acquired horizontally by a common ancestor of *Desulfitobacterium* spp. and *Desulfosporosinus* spp. from *Streptomyces coelicolor* or its ancestor. This would also offer a plausible explanation for the presence of *vanK_I_* in *Desulfitobacterium hafniense* Y51 and *Desulfosporosinus* sp. OT as a redundant reminiscence that has been lost from most *vanI*-containing strains due to lack of selection pressure.

It was investigated whether vancomycin could be used as a selective agent for purifying *Desulfitobacterium* spp. from enrichment cultures containing other, susceptible Gram-positive populations, such as *Sedimentibacter* spp., which have been repeatedly co-enriched with organohalide-respiring species of *Desulfitobacterium* and the closely related genus *Dehalobacter* (Van Doesburg *et al*., 2005; Bedard *et al*., 2006; Breitenstein *et al*., 2007; Sánchez-Andrea *et al*., 2012). A *D. hafniense* TCE1 culture, contaminated with a *Sedimentibacter* sp., was successfully purified using vancomycin as a selection agent. Searching public available meta/genome databases showed that *vanI* could not be found in any of the screened metagenomes but only in the genomes of the members of both *D. hafniense* or *chlororespirans* and the closely related *Desulfosporosinus* spp., supporting the hypothesis that *vanI*, unlike other vancomycin resistance clusters found in the gut microbiota, is not subject to frequent horizontal gene transfers. The use of vancomycin or teicoplanin may, thus, help in future isolation of new *Desulfitobacterium* or *Desulfosporosinus* spp. strains and may also be used as a convenient selection marker for genetic manipulations.

In conclusion, we have shown that *D. hafniense*, *D. chlororespirans* and *Desulfosporosinus* spp. contain a novel chromosomal gene cluster, designated *vanI*, conferring medium to high levels of vancomycin resistance, and medium level teicoplanin resistance. This feature can be both a useful intrinsic selection marker or a potential risk if mobilized into clinically relevant strains.

## Experimental procedures

### Strains and culturing conditions

The bacterial strains used in this study are listed in Table 1. *Escherichia coli* XL1-blue was grown in lysogeny broth at 37°C. *Desulfosporosinus* spp. were grown in DSMZ medium DSM 641 supplemented with 50 mM pyruvate for growth of *D. meridei*, or 12.5 mM pyruvate and 6.25 mM malate for growth of *D. youngia* or *D. orientis*.

*Desulfitobacterium* spp. were grown as 20 ml cultures in 120 ml serum bottles under an atmosphere of 100% N_2_. *Desulfitobacterium dichloroeliminans* DCA1 was grown as described previously (De Wildeman *et al*., 2003). *Desulfitobacterium metallireducens* was grown in the medium recommended by the DSMZ (DSM 838). All other strains of *Desulfitobacterium* spp. were cultivated in basal medium as described previously (Neumann *et al*., 1994), supplemented with 1 g/l yeast extract and with additional modifications from the original medium as follows.

After autoclaving, the medium was supplemented with vitamins and trace elements from anaerobic filter sterilized (0.22 μm pore size) stocks giving final concentrations per litre medium of 1.34 μM EDTA; 10.06 μM FeCl_2_●4H_2_O; 0.51 μM MnCl_2_●4H_2_O; 0.8 μM CoCl_2_●6H_2_O; 0.51 μM ZnCl_2_; 0.02 μM CuCl_2_; 0.04 μM AlCl_3_●6H_2_O; H_3_BO_3_; 0.15 μM Na_2_MoO_4_; 0.1 μM NiCl_2_●6H_2_O, 0.75 mM CaCl_2_●2H_2_O; 0.5 mM MgCl_2_●6H_2_O; 3 μg Na_2_SeO_3_; 9 μg Na_2_WO_4_●2H_2_O; 0.04 μl concentrated HCl; 50 μg biotin; 250 μg P-aminobenzoate; 50 μg pantothenate; 20 μg folic acid; 50 μg lipoic acid; 100 μg pyridoxine; 550 μg nicotinamide; 100 μg thiamine HCl; 50 μg riboflavin; 50 μg cyanocobalamine; and finally reducing buffer solutions to a final concentration of 1 mM Na_2_S●9H_2_O, 5.6 mM NH_4_HCO_3_ and 44.4 mM NaHCO_3_.

Cultures were routinely grown fermentatively on 40 mM pyruvate at the temperatures recommended by the DSMZ, unless stated otherwise. Pyruvate, lactate and PCE were added from sterile anaerobic stocks to final concentrations of 40, 20 and 10 mM, respectively, when indicated.

### Antibiotic susceptibility testing

All plating of *Desulfitobacterium* spp. and *Desulfosporosinus* spp. was performed in an anaerobic glove box containing an atmosphere of 96% N_2_ and 4% H_2_. Strains were streaked with cotton swaps, on plates prepared as described previously (Smidt *et al*., 1999), with a modification using Difco™ agar noble (Becton Dickinson, Le Pont de Claix, France) for solidification. The medium contained 40 mM pyruvate, 6 mM malate and 10 mM SO_4_ as carbon and energy source. Plates were prepared with various concentrations of vancomycin or teicoplanin (0, 1, 5, 10, 25, 50, 75, 150 and 250 μg/ml) and incubated anaerobically at 30°C.

### DNA isolation

Cells were harvested from 5 ml cultures by spinning at 6700 × *g* for 10 min. DNA was extracted from cell pellets using the Bio101 FASTDNA isolation kit according to manufacturers’ protocols (MP Biomedicals, Solon, OH, USA).

### PCR amplification, cloning and sequencing of *vanI* genes

All PCR reactions were performed using the GoTaq DNA Polymerase Kit (Promega, Leiden, The Netherlands) in a total reaction volume of 30 μl, with 0.5 μl DNA extract or cell lysate as template. PCR products were analysed on a 1% agarose gel using gene ruler 1 kb or 100 bp plus ladders (Fermentas, St. Leon-Rot, Germany).

A 987 bp fragment of the *vanI* gene was amplified using primers K46F/K47R (Supporting Information Table S2) and the following temperature programme: 94°C for 120 s, followed by 35 cycles of 94°C for 30 s, 56°C for 30 s and 72°C for 50 s, and a final elongation step at 72°C for 6 min. Resulting amplicons were purified prior to sequencing using the Zymo DNA Clean & Concentrator Kit (Zymo Research, San Francisco, CA, USA), sequenced in one direction using primer K46 and initially analysed using nucleotide blast (Altschul *et al*., 1990). An overlapping fragment of 512 bp was obtained for all amplicons, and used for phylogenetic analysis.

### Purity of *Desulfitobacterium hafniense* TCE1

Nearly full-length 16S rRNA gene fragments were amplified from *D. hafniense* TCE1 using primers 27F/1492R (Supporting Information Table S2) and the following temperature programme: 94°C for 120 s, followed by 35 cycles of 94°C for 30 s, 52°C for 40 s and 72°C for 90 s, ending with a final elongation step of 72°C for 5 min.

PCR products were purified as described above; cloning of 16S rRNA genes was done using the cloning vector pGEMT-Easy (Promega) and *E. coli* XL1-Blue as host following the manufacturer’s instructions. Full-length inserts were amplified from transformant cells, lysed by heating of 10 μl o/n culture in 90 μl TE (10 mM tris-HCl, 1 mM EDTA, pH 7.5) at 97°C for 10 min. PCR amplification using primers T7 and SP6, targeting the flanking region of the pGEMT-Easy cloning site (Supporting Information Table S2), was done as described above, using the following temperature programme: 94°C for 120 s, followed by 35 cycles of 94°C for 30 s, 52°C for 40 s and 72°C for 90 s, ending with a final elongation step of 72°C for 5 min.

Clones were analysed for the presence of *Desulfitobacterium* spp. derived 16S rRNA gene sequences using genus-specific primers DSB406F/619R (Supporting Information Table S2), and the following temperature programme: 94°C for 4 min, followed by 35 cycles of 94°C for 30 s, 58°C for 20 s, 72°C for 35 s, and a final elongation step of 72°C for 10 min. Inserts from three randomly selected clones that did not give a product with *Desulfitobacterium* specific primers were partially sequenced and analysed using blast (Altschul *et al*., 1990).

### DNA sequence analysis

Alignments of DNA sequences were done using muscle (Edgar, 2004), and trees constructed using the neighbour-joining method and tested by 1000 bootstraps using the mega5 software package (Tamura *et al*., 2011). Sequences obtained in this study were first aligned with those used in the study of Aminov and Mackie (2007) and one sequence representing the recently described cluster *vanM* (Xu *et al*., 2010). Finally, sequences obtained from genome databases were added (Markowitz *et al*., 2012a). Identity matrixes were generated using MatGAT with default settings (Campanella *et al*., 2003). Full genome alignment and analysis was done using the Artemis comparison tool, version 10 (Carver *et al*., 2005).

### Check for *vanI* homologues in (meta)genomes

All available (as of 4 February 2013; 1236 metagenomes covering a wide range of environments) metagenomes on the JGI IMG/M server (Markowitz *et al*., 2012b) were screened for the presence of *vanI* homologues. We performed an initial BlastP using the strictest possible cut-off, E < e-50, against the entire img/m database using VanI (dsy_3690) from *Desulfitobacterium hafniense* Y51 as query. We obtained 1338 sequences with homology to VanI, created a local blast database containing the contigs harbouring the corresponding DNA sequences, and performed a tblastx analysis against all contigs, using the *D. hafniense* Y51 *vanI* gene sequence as query. This was done in order to correct for genes that potentially have not been incorporated correctly into the BlastP database, due to for example frameshift mutations or assembly errors. The eight full-length d-Ala-d-Lac ligase gene homologues with the best hits to *vanI* (E ≤ 3.00E-135) were aligned and checked for phylogenetic affiliation with *vanI*.

### *vanI* homologues in sequenced bacterial genomes outside the *Desulfitobacterium* genus

All available genomes (as of 19 May 2014) at the JGI/IMG server (Markowitz *et al*., 2012a) were screened for the presence of *vanI* homologues. Initially, as for metagenome mining, BlastP search was done using *D. hafniense* Y51 VanI (dsy3690) sequence as query against all available genomes. This was followed by manual check of sequences giving strong (E = 0) hits to VanI. The *vanI* sequence identified in *Desulfosporosinus* sp. OT was found to be N-terminally truncated. A closer inspection of the genome sequence showed that this is due to either a frameshift mutation or sequencing error; by combining two reading frames, we were able to assemble a 1032 bp fragment. The corresponding predicted amino acid sequence showed 93.3% identity to the *vanI* homologue from *Desulfosporosinus youngiae* JW/Y JL-B18 (Supporting Information Table S1).

## Nucleotide sequence accession numbers

The obtained partial *vanI* D-Ala-D-Lac ligase gene sequences are available in The National Center for Biotechnology Information (NCBI) database under accession numbers HQ433580 to HQ433586.

## Supporting Information

Table S1 Identity matrix showing percentage amino acid identity of d-Ala-d-Lac ligases representing the currently known van gene clusters of the d-Ala-d-Lac type. The sequences used here are translated, using the standard bacterial genetic code, from the full-length genes used in Fig. 3. Accession numbers are given in parentheses. The identity matrix were generated using MatGAT as described under experimental procedures. Colour codes highlight the different vancomycin resistance clusters. Shades of brown indicates representatives of cluster II, whereas blue and purple indicates representatives of cluster I.

Table S2 Primers used in this study.
