# Supplementary material for: vanI: a novel d-Ala-d-Lac vancomycin resistance gene cluster found in Desulfitobacterium hafniense
Source: Microb Biotechnol. 2014 Jul 5;7(5):456–66. doi: 10.1111/1751-7915.12139 (PMC4229326; doi:10.1111/1751-7915.12139)
Supplement: Table S2 — Primers used in this study. [file mbt20007-0456-sd2.doc]

| **Name** | **Sequence 5’-3’** | **Target** | **Reference** |
| --- | --- | --- | --- |
| 27F | AGAGTTTGATCMTGGCTCAG | Bacterial 16s rRNA |  |
| 1492R | GGTTACCTTGTTACGACTT |  |
| DSB 406F | GTACGACGAAGGCCTTCGGT | 16s rRNA of desulfitobacteria |  |
| DSB619R | CCCAGGGTTGAGCCCTAGGT |
| T7 | TAATACGAACTCACTATAGG | T7 promoter | Promega |
| SP6 | GATTTAGGTGACACTATAG | SP6 promoter |
| K46F | AAGGCCAACGAGACAAGCC | *vanI* | This study |
| K47R | GCTGTTCCGAAGAACATCCCG |

**Supplemental table 2:** Primers used in this study
